# Building an early warning model for the risk of suicide attempt in people with depression based on machine learning: a single-centre study

**DOI:** 10.3389/fpsyt.2026.1856898

**Published:** 2026-08-14

**Authors:** Mian Zhang, Wen Liu, Wenzhi Pei, Long Chen, Min Pan, Anzhen Wang, Boyu Zhang, Xialong Cheng, Wen Xie

**Affiliations:** 1 School of Mental Health and Psychological Science, Anhui Medical University, Hefei, China; 2 Department of Child and Adolescent Psychiatry, Affiliated Psychological Hospital of Anhui Medical University, Hefei, China; 3 Department of Child and Adolescent Psychiatry, Hefei Fourth People's Hospital, Hefei, China; 4 Department of Psychology, Affiliated Psychological Hospital of Anhui Medical University, Hefei Fourth People's Hospital, Hefei, China

**Keywords:** attempted suicide, biomarkers, depression, machine learning, random forest, risk prediction

## Abstract

**Background:**

Attempted suicide is one of the most serious clinical consequences for patients with depression, and early identification of high-risk individuals is crucial for preventing suicide deaths. Traditional clinical assessment often relies on subjective judgment, lacking objective biological markers and multi-dimensional data integration analysis.

**Objective:**

This study aims to use machine learning algorithms to integrate demographic characteristics, clinical symptom scales, and blood biochemical indicators to construct and validate an early warning model for the risk of attempted suicide in patients with depression, and to explore key predictors.

**Methods:**

A total of 1,229 patients with depression were included in this study, including 578 cases in the suicide attempt group and 651 cases in the non-suicide attempt group. Demographic information, Hamilton Depression Scale (HAMD) sub-item scores, and biochemical indicators such as thyroid function, liver and kidney function, blood lipids, and electrolytes were collected. Predictive models were constructed using six machine learning algorithms: Random Forest (RF), Extreme Gradient Boost (XGBoost), Support Vector machine (SVM), multi-layer perceptron (MLP), logistic regression (LR), and Decision tree (DT). Model performance was evaluated by area (AUC) under the receiver operating characteristic curve (ROC), accuracy, sensitivity, specificity, and F1 score, and feature importance was explained by SHAP (Shapley Additive exPlanations) values.

**Results:**

Univariate analysis showed significant differences (P<0.05) between the two groups in terms of gender, education level, free triiodothyronine (FT3), albumin, triglycerides, blood potassium, social dysfunction, cognitive impairment, sense of hopelessness, and mental anxiety. In the comparison of model performance, random forest (RF) performed the best, with an AUC of 0.675 (95% CI: 0.621-0.727), outperforming other models. Feature importance analysis indicated that age of onset (AOO), body mass index (BMI), free thyroxine (FT3), and uric acid (UA) were core predictor variables consistent across models. The SHAP analysis further revealed that advanced age, low education level, high mental symptom markers, high cognitive impairment scores, and high uric acid levels significantly increased the risk of attempted suicide, while higher FT3 levels might have a protective effect.

**Conclusion:**

This study successfully constructed an early warning model for the risk of suicide attempt in patients with depression based on multimodal data. The random forest model demonstrated good predictive performance. The study found that in addition to traditional psychosocial factors, metabolic indicators such as BMI and uric acid and thyroid function (FT3) are also important risk predictors, providing a new biological perspective for early clinical intervention.

## 1 Introduction

**Figure 1:**
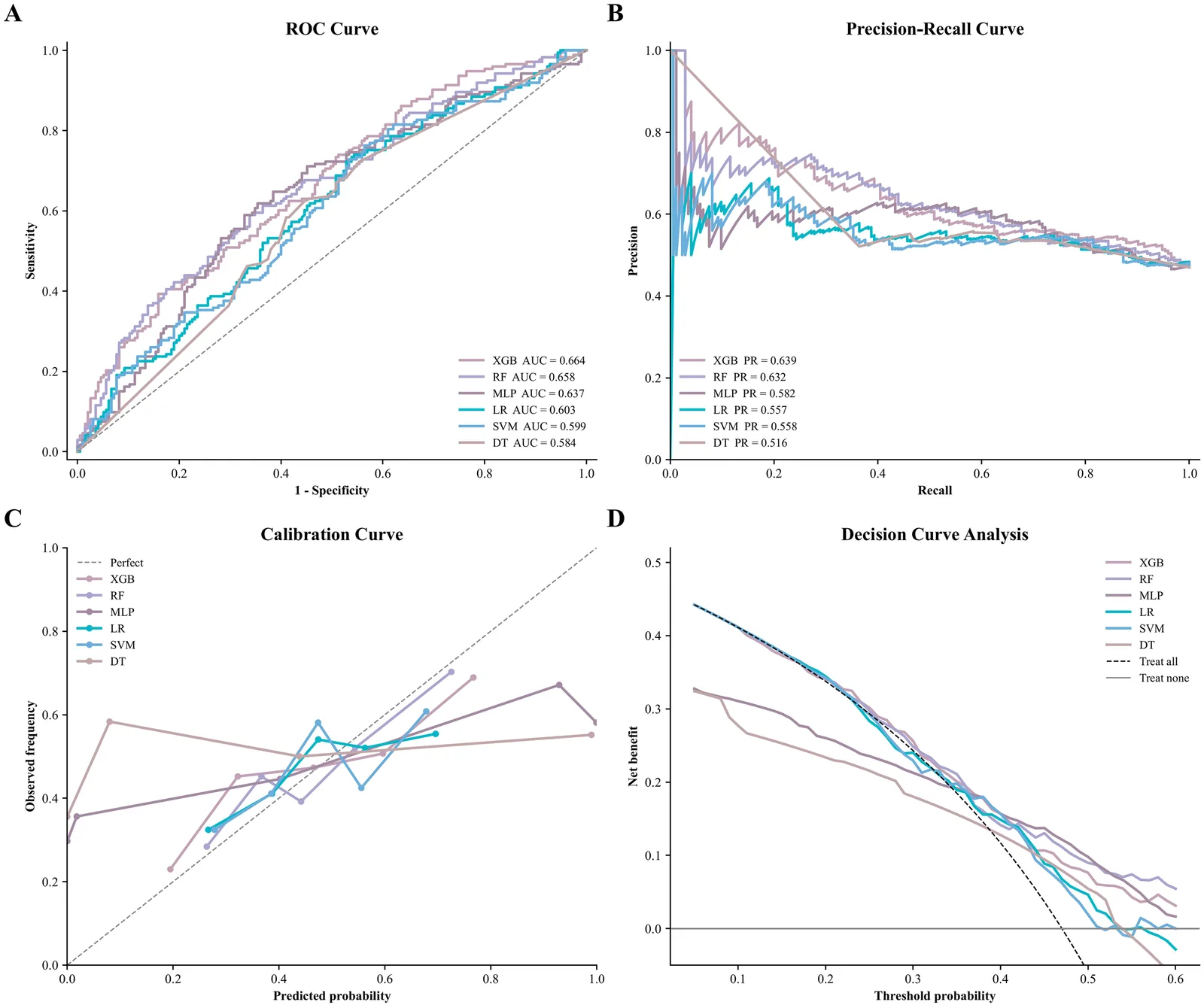
Discriminative and clinical performance of the six machine-learning models on the held-out test set. **(A)** Receiver operating characteristic (ROC) curves with the area under the curve (AUC) for each model. **(B)** Precision-recall curves. **(C)** Calibration curves showing the agreement between predicted probabilities and observed frequencies of a suicide attempt; the diagonal line denotes perfect calibration. **(D)** Decision curve analysis showing the net benefit of each model across a range of threshold probabilities, relative to the treat-all and treat-none strategies. RF, Random Forest; XGBoost, extreme gradient boosting; SVM, support vector machine; MLP, multilayer perceptron; LR, logistic regression; DT, decision tree.

Suicide is a major global public health challenge. According to the World Health Organization (WHO), nearly 800,000 people die by suicide each year, and the number of attempted suicides is dozens of times that figure (1). Depression is the strongest single predictor of suicidal behavior, with about half of those who die by suicide suffering from depression (2). Although antidepressants and psychotherapy have reduced the risk of suicide to some extent, the rate of attempted suicide among people with depression remains high and is often sudden and concealed (3). Therefore, developing efficient and accurate early warning tools to identify high-risk individuals and carry out targeted interventions is a key issue that urgently needs to be addressed in the field of psychiatry.

Traditional suicide risk assessment mainly relies on the interview experience of clinicians and standardized psychological scales (such as the Baker Despair Scale, the Columbia Suicide Severity Scale, etc.) (4). However, these methods have obvious limitations: on the one hand, they are highly dependent on subjective reports from patients, and patients in crisis tend to conceal suicidal thoughts; On the other hand, traditional statistical methods, such as linear regression, struggle to capture complex nonlinear relationships and high-dimensional interactions among variables (5). In recent years, with the rapid development of artificial intelligence technology, Machine Learning (ML) has shown great potential in the diagnosis and prognosis prediction of mental disorders. Multiple studies have shown that ensemble learning algorithms (such as random forest, XGBoost) outperform traditional statistical models in handling heterogeneous medical data and can more accurately mine potential risk patterns (6–8).

In addition to psychosocial factors, there is growing evidence suggesting that biological indicators play a significant role in suicidal behavior. Dysfunction of the hypothalamic-pituitary-thyroid (HPT) axis is closely associated with the severity of depression and suicide risk, and low levels of thyroid hormones have been proven to be associated with impulsivity and suicidal behavior (9–11). In addition, chronic inflammatory responses, oxidative stress, and metabolic disorders (such as dyslipidemia and changes in uric acid levels) are also believed to be involved in the pathophysiological mechanisms of suicidal behavior (12–15). For example, a decrease in the level of uric acid, an endogenous antioxidant, may reflect an increase in oxidative stress, which in turn is associated with increased neurotoxicity and suicide risk (16, 17). However, most current studies focus only on indicators in a single domain and lack systematic research that integrates demographic, clinical symptoms, and multi-dimensional biochemical indicators.

In view of this, this study aims to use large samples of clinical data, integrate demographic characteristics, detailed clinical symptom dimensions, and comprehensive blood biochemical indicators, and apply multiple mainstream machine learning algorithms to construct an early warning model for the risk of attempted suicide in patients with depression. Not only did we compare the predictive performance of different algorithms, but we also used explainable artificial intelligence technology (SHAP) to deeply analyze the direction and intensity of key predictors. This study aims to provide clinicians with objective auxiliary decision-making tools by discovering new biological markers and constructing high-precision predictive models, thereby achieving early identification and precise intervention of suicide risk.

## 2 Methods

### 2.1 Study design and participants

This retrospective study was conducted at a specialized psychiatric hospital. We enrolled 1,229 inpatients who met the DSM-5 criteria for major depressive disorder (MDD). Patients were classified into a suicide-attempt group (n = 578) and a non-attempt group (n = 651), according to whether a suicide attempt occurred during hospitalization. Exclusion criteria were comorbid severe neurological disorders, intellectual disability, or incomplete medical records. This study has been approved by the Ethics Committee of Hefei Fourth People’s Hospital in Anhui Province, China (Approval Number: HFSY-IRB-YJ-KYXM-CL (2024–064–001)). As the study adopts a retrospective and anonymized design, written informed consent is not required.

### 2.2 Data collection

Clinical data were extracted from electronic medical records at admission and covered three domains. Sociodemographic variables included sex, age, marital status, educational level (low or high), body weight, and body mass index (BMI). Clinical variables comprised age of onset (AOO), illness duration, family history of psychiatric disorders, first-episode status, somatic symptoms, psychotic symptoms, and social functioning deficits. Blood biomarkers covered thyroid function (T3, T4, TSH, FT3, FT4), liver function (total bilirubin, direct bilirubin, total protein, albumin, globulin, alanine aminotransferase, aspartate aminotransferase, gamma-glutamyl transferase, total bile acids), lipid profile (total cholesterol, high-density lipoprotein cholesterol, triglycerides, apolipoprotein A1, apolipoprotein B), kidney function (blood urea nitrogen, creatinine, uric acid), electrolytes (potassium, sodium, chloride, calcium, magnesium, inorganic phosphate), creatine kinase, and glucose. Psychopathology was quantified with factor scores from the Hamilton Depression Rating Scale (anxiety/somatization, weight loss, cognitive disturbance, diurnal variation, retardation, sleep disturbance, and hopelessness) and the Hamilton Anxiety Rating Scale (somatic and psychic anxiety).

### 2.3 Data preprocessing and feature selection

Variables with more than 20% missing values were excluded. The cohort was first split into a training set (70%) and a test set (30%) by stratified random sampling on the outcome, with a fixed random seed. All preprocessing parameters were derived from the training set only. Missing continuous values were imputed with the training-set median, which was applied to both sets; categorical variables had no missing values. Continuous predictors were standardized by z-score, with the mean and standard deviation estimated from the training set and applied to the test set.

Feature selection used the least absolute shrinkage and selection operator (LASSO) with a binomial logistic model. The penalty parameter was tuned by 10-fold cross-validation on the training set, and the value defined by the one-standard-error rule was used. Predictors with non-zero coefficients were retained for model development.

### 2.4 Machine learning model development and evaluation

Six algorithms were trained and compared: Random Forest (RF), extreme gradient boosting (XGBoost), support vector machine (SVM), multilayer perceptron (MLP), logistic regression (LR), and decision tree (DT). The models were implemented in Python with the scikit-learn and XGBoost libraries and used the LASSO-selected features. For each algorithm, hyperparameters were optimized by grid search with 10-fold cross-validation on the training set, using AUC as the selection criterion; the search space is summarized in **Supplementary Table 1**. No class-rebalancing or oversampling was applied to the training set.

Model performance was assessed on the held-out test set in terms of discrimination, calibration, and clinical utility. Discrimination was measured by AUC with 95% confidence intervals from 2,000 bootstrap resamples, accuracy, sensitivity, specificity, positive predictive value (PPV), negative predictive value (NPV), and F1 score. Calibration was measured by calibration curves, the Brier score, and the calibration slope and intercept. Clinical utility was measured by decision curve analysis across a range of threshold probabilities. The best-calibrated model was designated as the primary model for interpretation.

### 2.5 Model interpretability and statistical analysis

Model interpretation used SHapley Additive exPlanations (SHAP). Interventional TreeSHAP was applied to the primary model on the test set, yielding feature contributions on the probability scale. Global feature importance was displayed with a beeswarm summary plot, and individual feature effects with dependence plots.

Baseline characteristics were compared between the two groups using independent-samples t-tests for continuous variables and chi-square tests for categorical variables. P-values for the continuous variables were adjusted for multiple comparisons with the Benjamini-Hochberg false discovery rate procedure. All tests were two-tailed, and P < 0.05 was considered statistically significant. Feature selection was performed in R (version 4.6.0) with the glmnet and caret packages; model development, evaluation, and interpretation were performed in Python (version 3.14) with scikit-learn (1.9.0), XGBoost (3.3.0), and SHAP (0.52.0).

## 3 Results

### 3.1 Baseline characteristics

A total of 1,229 patients with MDD were analyzed, comprising 578 with a suicide attempt and 651 without (**Table 1**). Among sociodemographic variables, the suicide-attempt group had a higher proportion of women (73.9% vs. 62.2%, P < 0.001) and of patients with low educational level (72.7% vs. 61.6%, P < 0.001); age and BMI did not differ between groups. Of the 44 continuous variables, ten differed at the nominal level (P < 0.05). After Benjamini-Hochberg correction, three remained significant: FT3 (3.780 ± 1.105 vs. 3.989 ± 1.128, q = 0.021) and albumin (41.944 ± 3.781 vs. 42.820 ± 4.516, q = 0.021) were lower in the suicide-attempt group, and cognitive-disturbance scores were higher (5.668 ± 3.545 vs. 4.869 ± 3.459, q = 0.004). The nominal differences in the remaining markers, including triglycerides, potassium, social functioning, hopelessness, and psychic anxiety, did not survive correction.

**Table 1:** Comparison of characteristics between suicide attempters and non-attempters (n=1229).

Characteristic	No suicide attempt (*n* = 651)	Suicide attempt (*n* = 578)	*χ²/t*	*P*
Sex			18.516	<0.001
Male	246 (37.8%)	151 (26.1%)		
Female	405 (62.2%)	427 (73.9%)		
Family history			0.000	1.000
No	601 (92.3%)	533 (92.2%)		
Yes	50 (7.7%)	45 (7.8%)		
First episode			3.162	0.075
No	373 (57.3%)	301 (52.1%)		
Yes	278 (42.7%)	277 (47.9%)		
Body symbols			0.301	0.583
No	285 (43.8%)	263 (45.5%)		
Yes	366 (56.2%)	315 (54.5%)		
Mental symbols			0.588	0.443
No	525(80.6%)	455(78.7%)		
Yes	126(19.4%)	123(21.3%)		
Education			16.413	<0.001
Low	401(61.6%)	420(72.7%)		
High	250(38.4%)	158(27.3%)		
Age	41.710 ± 13.910	40.285 ± 16.172	-1.660	0.357
BMI	23.047 ± 4.315	22.903 ± 4.378	-0.580	0.807
AOO	36.544 ± 13.532	36.014 ± 15.725	-0.635	0.807
Disease course	5.723 ± 7.285	5.389 ± 7.243	-0.805	0.741
T3	1.358 ± 0.415	1.308 ± 0.404	-2.068	0.190
T4	50.028 ± 51.147	44.380 ± 49.430	-1.922	0.219
TSH	2.136 ± 1.616	2.156 ± 2.053	0.184	0.963
FT3	3.989 ± 1.128	3.780 ± 1.105	-3.200	0.021
FT4	7.761 ± 7.508	6.821 ± 7.194	-2.186	0.182
Total bilirubin	11.793 ± 6.613	11.703 ± 6.238	-0.219	0.963
Direct bilirubin	4.686 ± 2.557	4.767 ± 2.576	0.499	0.815
Total protein	64.606 ± 8.937	64.860 ± 7.327	0.483	0.815
Albumin	42.820 ± 4.516	41.944 ± 3.781	-3.269	0.021
Globulin	23.379 ± 4.347	23.403 ± 3.713	0.094	0.976
Alanine aminotransferase	20.166 ± 20.650	19.296 ± 18.538	-0.693	0.796
Aspartate aminotransferase	18.043 ± 14.415	18.474 ± 13.332	0.487	0.815
Gamma-glutamyl transferase (GGT)	24.231 ± 25.404	24.086 ± 29.187	-0.084	0.976
Total bile acids	4.075 ± 3.819	3.740 ± 2.819	-1.534	0.424
Total cholesterol	4.372 ± 1.083	4.338 ± 0.956	-0.570	0.807
High density lipoprotein	1.308 ± 0.454	1.287 ± 0.361	-0.876	0.729
Triglycerides	1.425 ± 1.176	1.293 ± 0.764	-2.267	0.173
Apolipoprotein A	1.305 ± 0.633	1.278 ± 0.259	-0.847	0.729
Apolipoprotein B	0.810 ± 0.459	0.827 ± 0.401	0.615	0.807
Urea nitrogen	4.541 ± 1.564	4.647 ± 1.555	1.078	0.729
Creatinine	62.162 ± 16.525	62.195 ± 18.907	0.030	0.976
Uric acid	289.486 ± 93.694	284.514 ± 88.750	-0.937	0.729
Potassium	3.913 ± 0.383	3.850 ± 0.363	-2.678	0.083
Sodium	144.004 ± 53.867	141.633 ± 3.488	-0.911	0.729
Chlorine	105.155 ± 2.683	105.202 ± 2.926	0.261	0.963
Calcium	2.321 ± 0.192	2.331 ± 0.252	0.753	0.764
Creatine kinase	115.367 ± 466.244	116.872 ± 356.180	0.056	0.976
Glucose	5.115 ± 0.962	5.232 ± 1.925	1.277	0.633
Magnesium	0.897 ± 0.071	0.904 ± 0.167	0.906	0.729
Inorganic Phosphate	1.201 ± 0.169	1.203 ± 0.172	0.197	0.963
Social functioning deficits	9.885 ± 4.601	10.452 ± 4.672	2.127	0.185
Anxiety	7.822 ± 3.576	7.559 ± 3.809	-1.238	0.633
Weight loss	0.770 ± 0.900	0.750 ± 0.850	-0.395	0.871
Cognitive disturbances	4.869 ± 3.459	5.668 ± 3.545	3.953	0.004
Diurnal Variation	0.948 ± 1.029	0.889 ± 1.096	-0.959	0.729
Retardation	7.846 ± 2.253	7.958 ± 2.272	0.857	0.729
Sleep disturbances	5.270 ± 2.137	5.288 ± 2.096	0.146	0.973
Hopelessness	6.667 ± 2.815	7.086 ± 2.824	2.578	0.088
Somatic anxiety	6.989 ± 4.884	7.267 ± 5.254	0.952	0.729
Psychic anxiety	12.859 ± 4.154	13.381 ± 4.936	1.994	0.204

Data are presented as mean ± SD (continuous variables) or n (%) (categorical variables). Between-group comparisons used independent-samples t-tests for continuous variables and Pearson χ² tests for categorical variables. For continuous variables, P values were adjusted for multiple comparisons using the Benjamini–Hochberg false-discovery-rate procedure (across the 44 continuous variables); P values for categorical variables are unadjusted. Abbreviations: BMI, body mass index; AOO, age of onset; T3, triiodothyronine; T4, thyroxine; TSH, thyroid-stimulating hormone; FT3, free triiodothyronine; FT4, free thyroxine; GGT, gamma-glutamyl transferase.

### 3.2 Model performance

Discrimination on the held-out test set was modest and similar across models, with overlapping confidence intervals (**Table 2**, **Figure 1**). XGBoost achieved the highest AUC (0.664, 95% CI 0.610-0.719), closely followed by Random Forest (0.658, 0.603-0.713). MLP, logistic regression, SVM, and decision tree reached AUCs of 0.637, 0.603, 0.599, and 0.584, respectively. Calibration separated the models more clearly. Random Forest was the best-calibrated model (calibration slope 0.81, Brier score 0.230), whereas XGBoost was over-confident (slope 0.64) and MLP and decision tree were poorly calibrated (slopes 0.07 and 0.03). Decision curve analysis showed a positive net benefit for the better-calibrated models over the treat-all and treat-none strategies across the low-to-moderate threshold range. Given comparable discrimination and its superior calibration, Random Forest was selected as the primary model.

**Table 2:** Performance analysis of the models.

Model	AUC	95% CI	ACC	Sensitivity	Specificity	PPV	NPV	F1
RF	0.658	0.603-0.713	0.620	0.497	0.728	0.619	0.620	0.551
XGBoost	0.664	0.610-0.719	0.606	0.566	0.641	0.583	0.625	0.575
SVM	0.599	0.542-0.657	0.549	0.468	0.621	0.523	0.568	0.494
MLP	0.637	0.578-0.693	0.628	0.601	0.651	0.605	0.648	0.603
LR	0.603	0.546-0.663	0.576	0.509	0.636	0.553	0.593	0.530
DT	0.584	0.529-0.641	0.587	0.584	0.590	0.558	0.615	0.571

Metrics were calculated on the independent test set; the 95% CI for AUC was estimated with 2000 bootstrap resamples. AUC, area under the receiver operating characteristic curve; CI, confidence interval; ACC, accuracy; PPV, positive predictive value; NPV, negative predictive value; F1, F1 score. RF, Random Forest; XGBoost, Extreme Gradient Boosting; SVM, Support Vector Machine; MLP, Multi-Layer Perceptron; LR, Logistic Regression; DT, Decision Tree.

### 3.3 Feature selection and feature importance

**Figure 2:**
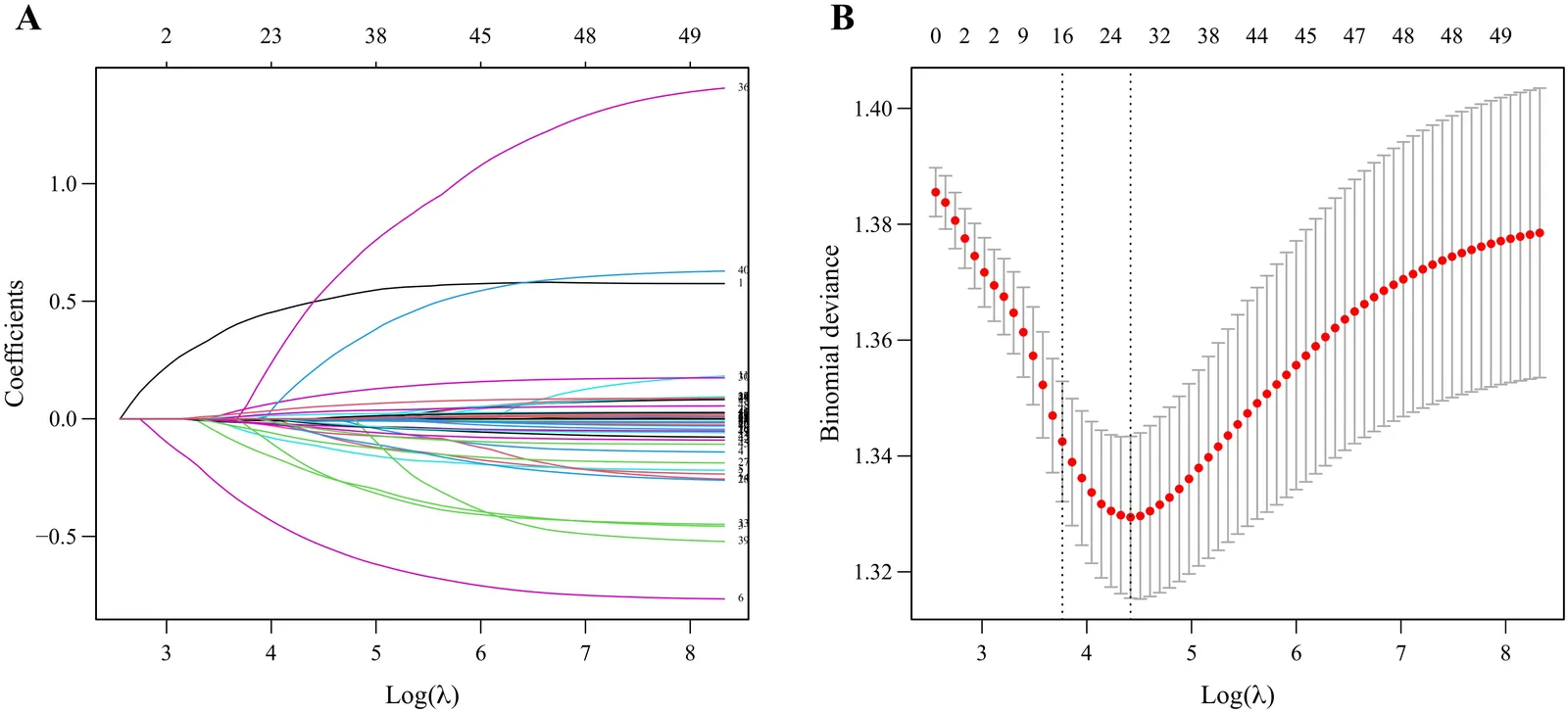
Feature selection by the least absolute shrinkage and selection operator (LASSO). **(A)** Coefficient profiles of the candidate predictors as a function of the log-transformed penalty parameter, log(lambda). **(B)** Ten-fold cross-validation curve of the binomial deviance versus log(lambda); the left dashed line marks the lambda with minimum deviance and the right dashed line the lambda selected by the one-standard-error rule, at which 16 predictors were retained.

LASSO retained 16 predictors from the candidate variables (**Figure 2**; one-standard-error penalty, λ = 0.023). Ranked by absolute coefficient, the strongest predictors were female sex and educational level, followed by first-episode status, calcium, psychotic symptoms, blood urea nitrogen, triglycerides, potassium, cognitive disturbance, albumin, anxiety, total bile acids, FT3, hopelessness, social functioning, and age. These 16 predictors were carried forward to model development.

### 3.4 Model interpretability and key predictor analysis

**Figure 3:**
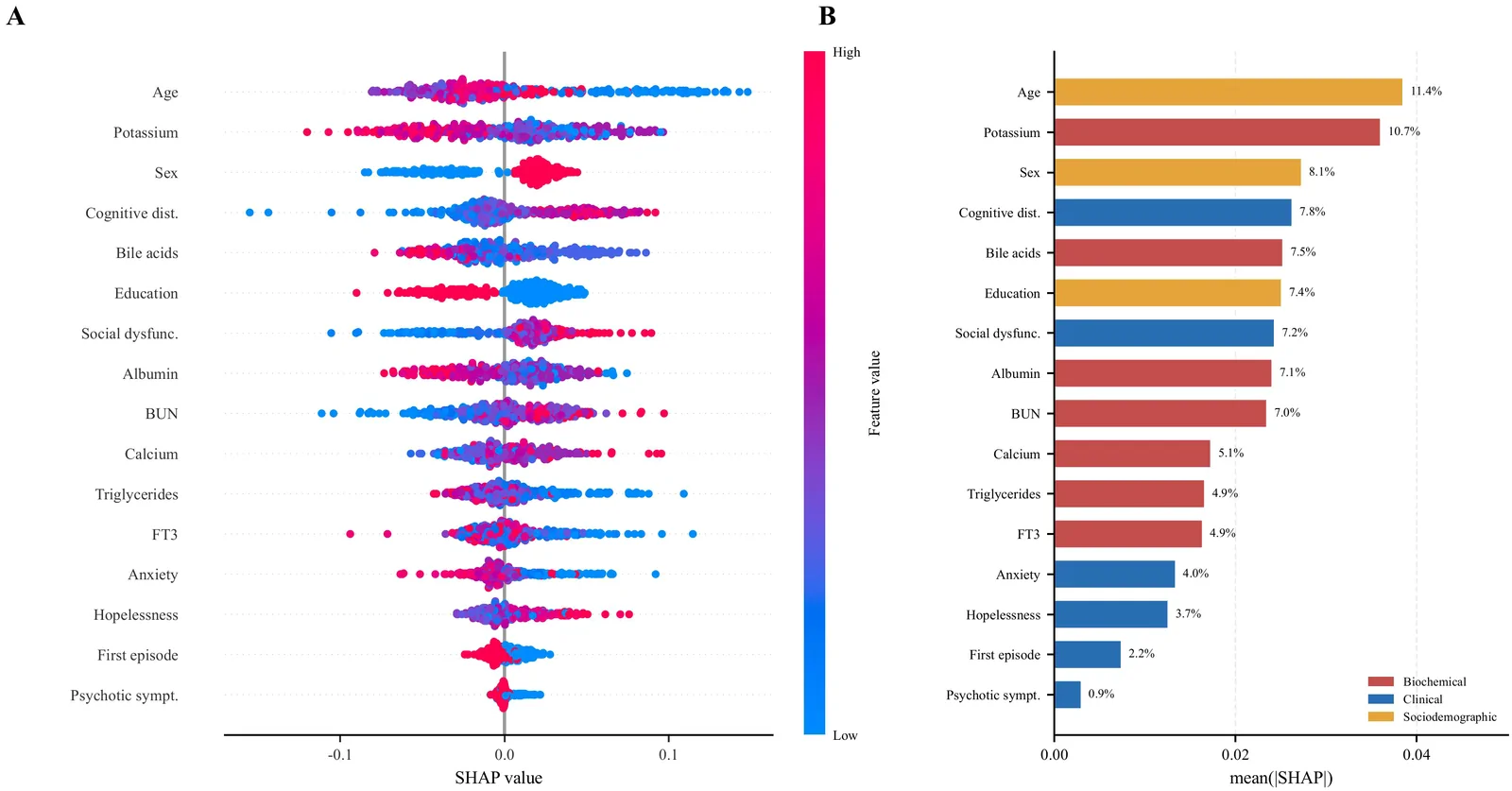
SHAP summary for the Random Forest model on the test set. **(A)** Beeswarm plot: each point represents one patient, positioned along the horizontal axis by its SHAP value (impact on the predicted probability of a suicide attempt) and colored by the feature value (blue, low; red, high); features are ordered from top to bottom by decreasing mean absolute SHAP value. **(B)** Bar plot ranking the features by mean absolute SHAP value. SHAP, SHapley Additive exPlanations.

**Figure 3** presents the SHAP summary for the Random Forest. In the beeswarm plot (**Figure 3A**) each point is a single patient, positioned along the horizontal axis by its SHAP value and colored by the value of the feature (blue low, red high); the bar plot (**Figure 3B**) ranks the features by mean absolute SHAP value. The five most influential predictors were age (11.4%), potassium (10.7%), sex (8.1%), cognitive disturbance (7.8%), and total bile acids (7.5%), followed by educational level, social functioning, albumin, and blood urea nitrogen.

**Figure 4:**
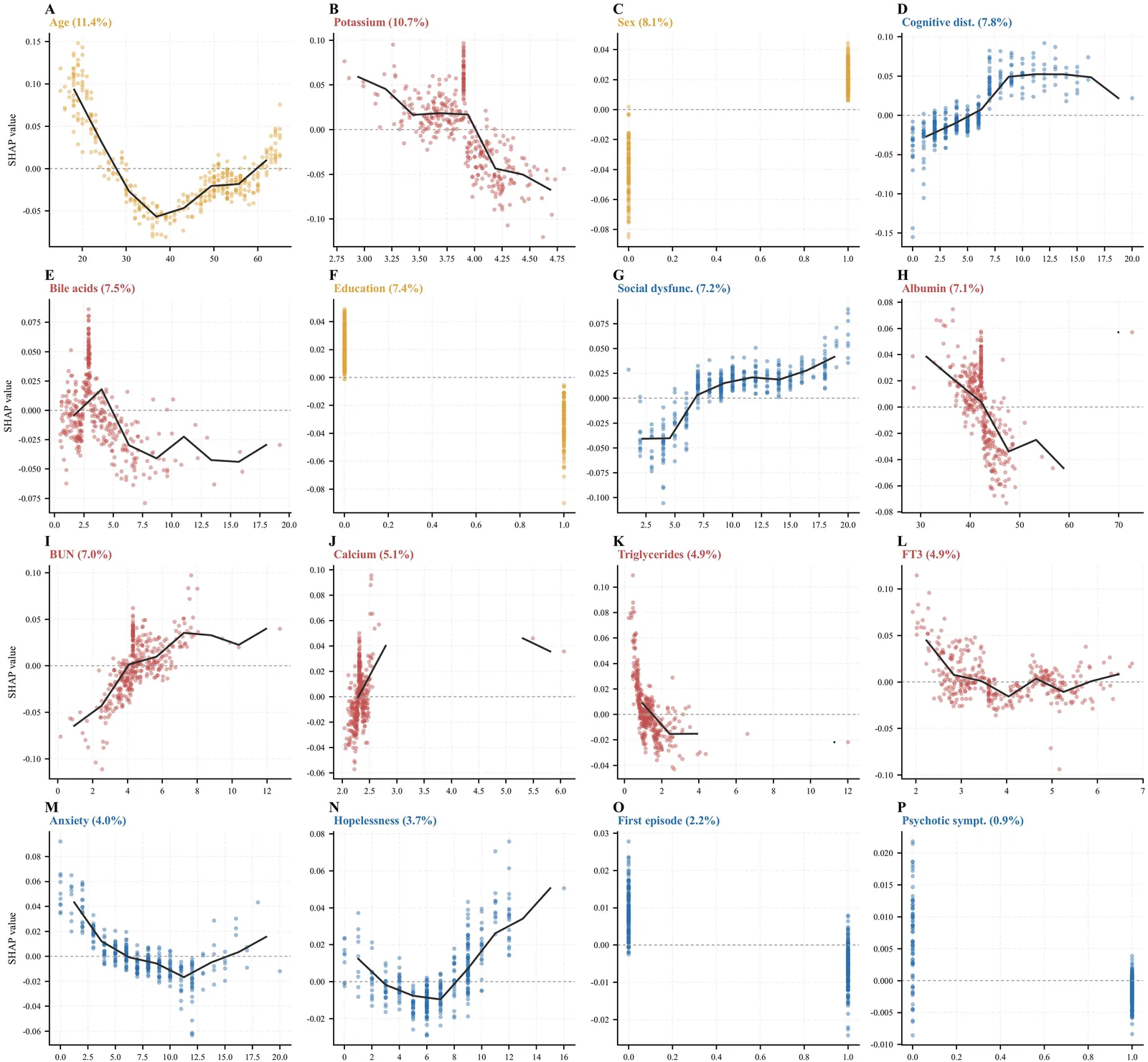
SHAP dependence plots for the 16 model predictors [panel **(A-P)**], ordered by decreasing mean absolute SHAP value. In each panel, the horizontal axis is the feature value and the vertical axis is the corresponding SHAP value across patients in the test set; a positive SHAP value indicates an increase, and a negative value a decrease, in the predicted probability of a suicide attempt.

The dependence plots (**Figure 4**) show how each feature related to the predicted risk. Higher predicted risk was associated with female sex, higher cognitive-disturbance and social-functioning-deficit scores, higher blood urea nitrogen, and younger age; lower predicted risk was associated with higher educational level, potassium, albumin, FT3, and total bile acids. Several continuous predictors showed non-linear relationships.

Across the analyses, the suicide-attempt group was characterized by female sex, low educational level, lower FT3 and albumin, and higher cognitive disturbance; discrimination was comparable across the six models (AUC 0.58-0.66), Random Forest showed the best calibration, and SHAP ranked age, potassium, sex, cognitive disturbance, and total bile acids as the leading contributors to individual risk.

## 4 Discussion

This study utilized machine learning techniques and integrating multi-dimensional clinical data, successfully constructed an early warning model for the risk of attempted suicide in patients with depression. The results showed that the random forest model performed best in differentiating suicide attempters from those without suicide attempts (AUC = 0.675), and identified a set of key predictors that were stable across the model, including age of onset, BMI, FT3, and uric acid. This finding not only validates the core position of psychosocial factors, but also highlights the potential value of metabolic and endocrine indicators in suicide risk assessment.

Our results are consistent with a large number of previous studies, confirming that cognitive impairment, feelings of hopelessness, and psychogenic anxiety are strong predictors of attempted suicide (18, 19). The SHAP analysis showed that the more severe the cognitive dysfunction, the higher the risk of suicide, possibly because cognitive rigidity limits patients’ alternative solutions to problems and makes them more likely to choose extreme means (20). In addition, male gender and low educational attainment showed a higher risk in this study, which is consistent with some epidemiological findings suggesting that low socioeconomic status and gender-specific coping styles (such as men being more inclined to use lethal means) may play a significant role in suicidal behavior (21).

One notable finding of this study is that a reduction in FT3 levels is significantly associated with an increased risk of attempted suicide. Thyroid hormones are crucial for the development and functional maintenance of the central nervous system, and hypothyroxine status is often accompanied by aggravated depression, decreased cognitive function, and increased impulsivity (22). Previous studies have shown that hypothyroidism is an independent risk factor for treatment-resistant depression and suicidal behavior, and thyroid hormone supplementation can sometimes be used as a synergistic treatment for treatment-resistant depression (23, 24). Our model further quantifies this relationship, suggesting that thyroid function, especially FT3 levels, should be routinely monitored in clinical practice when assessing suicide risk, and high vigilance should be given to those with low levels.

The findings regarding uric acid and BMI are worthy of further exploration. The traditional view often holds that high uric acid is a cardiovascular risk factor, but in the neuropsychiatric field, reduced levels of uric acid, as a major endogenous antioxidant, are typically associated with oxidative stress-mediated nerve damage, which in turn is linked to depression and suicide (15). However, in this study, high uric acid was instead associated with high risk (see SHAP analysis), which may be related to the specificity of the sample (such as coexisting metabolic syndrome, dietary structure or drug effects), or a manifestation of a non-linear relationship (i.e., both too low and too high are unfavorable). Similarly, high BMI is often regarded as a health risk in the general population, but in patients with depression, weight changes are complex, and high BMI may reflect specific inflammatory states or lifestyle disorders, all of which require further clarification in future studies (25–27).

Compared to traditional logistic regression, ensemble learning algorithms (such as RF, XGBoost) can better handle nonlinear interactions between variables. For example, the study found that men had a more significant increase in risk under a high mental symptom load, an interaction effect that is easily overlooked in linear models (28). Although the AUC of the current model is not yet perfect, it has great potential as an auxiliary screening tool. In the future, such models could be embedded in electronic medical record systems to automatically calculate risk scores when patients enter relevant indicators, alerting doctors to conduct more in-depth psychological assessments.

Future research should focus on expanding the sample size and incorporating multi-center data to enhance the generalization ability of the model. At the same time, longitudinal tracking designs should be introduced to explore the time series relationship between dynamic changes in biochemical indicators and suicidal behavior. In addition, the combination of multimodal data such as genomics, neuroimaging, and digital phenotypes are expected to further enhance predictive accuracy and advance psychiatric diagnosis and treatment towards precision medicine (29).

## 5 Limitations

Although this study constructed a model with certain predictive value, the following limitations still exist. This study was a cross-sectional survey and could not establish a causal relationship between variables and attempted suicide. For example, changes in biochemical indicators may be a result rather than a cause of suicidal behavior, or a side effect of long-term drug treatment. Data coming only from a single mental health center may have selection bias, limiting the model’s external validity (generalization ability) in other regions or at different levels of medical care.

The study relied on retrospective medical records to define “attempted suicide”, and there may be underreporting or inaccurate classification, especially for minor self-harm behaviors that did not seek medical help. The study did not adequately control for patients’ medication use before admission, and different types of antidepressants or mood stabilizers may interfere with biochemical indicators such as blood lipids and thyroid function, thereby affecting the authenticity of model characteristics.

While the random forest model performs fairly well in similar studies (AUC 0.675), it still falls short of the high precision required for wide clinical application (typically AUC > 0.80). This may result from the lack of inclusion of genetic data, neuroimaging data, or a more refined assessment of life event stress.

To sum up, this study confirms the feasibility of machine learning-based multimodal models in predicting the risk of attempted suicide in patients with depression. In addition to focusing on traditional psychological symptoms, clinicians should pay attention to patients’ thyroid function and metabolic indicators for a more comprehensive risk assessment and early intervention.

## Data Availability

The raw data supporting the conclusions of this article will be made available by the authors, without undue reservation.
