# Supplementary material for: Building an early warning model for the risk of suicide attempt in people with depression based on machine learning: a single-centre study
Source: Front Psychiatry. 2026 Aug 14;17:1856898. doi: 10.3389/fpsyt.2026.1856898 (PMC13522196; doi:10.3389/fpsyt.2026.1856898)
Supplement: Supplementary file 1 [file Table1.docx]

**Supplementary Table S1.** Hyperparameter search space and optimal values for machine learning models.

| Model | Hyperparameter | Search Space | Optimal Value |
| --- | --- | --- | --- |
| RF | n_estimators | 100, 200, 300 | 100 |
|  | max_depth | None, 10, 20, 30 | None |
|  | min_samples_split | 2, 5, 10 | 5 |
| XGBoost | n_estimators | 100, 200, 300 | 200 |
|  | max_depth | 3, 5, 7 | 3 |
|  | learning_rate | 0.01, 0.05, 0.1 | 0.05 |
| SVM | C | 0.1, 1, 10, 100 | 1 |
|  | gamma | scale, auto, 0.01, 0.1 | 0.01 |
| MLP | hidden_layer_sizes | (100, 50), (50, 25), (100, 100, 50) | (100, 100, 50) |
|  | alpha | 0.0001, 0.001, 0.01 | 0.0001 |
|  | activation | relu, tanh | relu |
|  | learning_rate | constant, adaptive | constant |
| LR | C | 0.01, 0.1, 1, 10, 100 | 0.1 |
|  | solver | lbfgs, liblinear | lbfgs |
| DT | max_depth | 5, 10, 20, None | 10 |
|  | min_samples_split | 2, 5, 10 | 2 |
|  | criterion | gini, entropy | gini |

**Note:** RF, Random Forest; XGBoost, Extreme Gradient Boosting; SVM, Support Vector Machine; MLP, Multi-Layer Perceptron; LR, Logistic Regression; DT, Decision Tree. Hyperparameters were tuned by grid search with 10-fold cross-validation (scoring = AUC) on the training set.
